# Prognostic role of *DFNA5* in head and neck squamous cell carcinoma revealed by systematic expression analysis

**DOI:** 10.1186/s12885-021-08692-w

**Published:** 2021-08-25

**Authors:** Zhiguo Liu, Hongyan Liu, Qian Dong, Hongyu Li, Bin Zhang, Yufeng Liu, Limei Zhong, Haikuo Tang

**Affiliations:** 1 Department of Oral and Maxillofacial Surgery, Hospital of Stomatology, Sun Yat-sen University, 56 Lingyuanxi Road, Guangzhou, 510055 China; 2 Guangdong Province Key Laboratory of Stomatology, Guangzhou, China; 3 Department of Operative Dentistry and Endodontics, Hospital of Stomatology, Sun Yat-Sen University, 56 Lingyuanxi Road, Guangzhou, 510055 China; 4 Guangzhou First People’s Hospital, Guangzhou, 510000 China; 5 Department of Laboratory Medicine, Guangdong Second Provincial General Hospital, No. 466 Xingang Middle Road, Haizhu District, Guangzhou, 510317 China

**Keywords:** *DFNA5*, Head and neck cancer, Survival prognosis, Lymphocyte infiltration

## Abstract

**Background:**

The gasdermin E gene (*GSDME*, also known as *DFNA5*) is mutated in familial aging-related hearing loss. Recent studies have also revealed that the expression of *DFNA5* is suppressed in many cancer types; however, little is known about the function of *DFNA5* in head and neck squamous cell carcinoma (HNSCC). Accordingly, the aim of the present study was to evaluate the expression of *DFNA5* and explore its prognostic value in HNSCC.

**Result:**

We used a set of bioinformatics tools, including Oncomine, TIMER, TISIDB, cBioPortal, and GEPIA, to analyze the expression of *DFNA5* in patients with HNSCC from public databases. Kaplan-Meier plotter was used to evaluate the potential prognostic significance of *DFNA5*. *DFNA5* mRNA levels were significantly higher in HNSCC tissues than in normal tissues, and high *DFNA5* expression was correlated with worse survival. Gene Ontology and Kyoto Encyclopedia of Genes and Genomes pathway analyses showed that *DFNA5* expression has a strong positive correlation with cell adhesion and the integrin signaling pathway, whereas its expression was negatively correlated with the levels of infiltrating B cells (cor = − 0.223, *P* = 8.57e-07) and CD8 T cells (cor = − 0.223, *P* = 2.99e-07).

**Conclusion:**

This study demonstrates that *DFNA5* expression has prognostic value for HNSCC patients. Moreover, these results suggest that regulation of lymphocyte infiltration is the mechanism underlying the function of *DFNA5* in HNSCC.

**Supplementary Information:**

The online version contains supplementary material available at 10.1186/s12885-021-08692-w.

## Introduction

Head and neck cancers include a wide variety of cancers varying in location and histological types. One of the subcategories is head and neck squamous cell carcinomas (HNSCCs), which include tumors of the nasal cavity, nasopharynx, oral cavity, oropharynx, hypopharynx, and larynx [1, 2]. Approximately 600,000 new cases of HNSCC are diagnosed each year worldwide, often at an advanced stage. Moreover, epidemiological data indicate that the incidence of oral cancer is increasing year after year [3]. Although the use of combination therapy (i.e., surgical techniques, chemotherapy, and radiation therapy) has significantly improved local control and the overall quality of life of HNSCC patients, their survival rate has only increased slightly in the past 20 years [4]. Therefore, it is imperative to understand the potential molecular carcinogenic pathways of HNSCC, which is expected to identify markers that can help to improve the diagnosis, treatment, and prevention of the disease.

Gasdermin E (*GSDME*), also known as deafness autosomal dominant 5 (*DFNA5*), was identified as a gene involved in an autosomal dominant form of inherited hearing impairment in 1998 [5]. Interestingly, subsequent reports showed that *DFNA5* also plays a role in tumor biology. Specifically, *DFNA5* has been suggested to act as a tumor suppressor, since it was shown to inhibit the colony formation and cell proliferation of gastric cancer, melanoma, and colorectal cancer cells, and could also suppress the aggressive behavior of breast cancer [6]. Recently, some chemotherapeutic agents such as cisplatin [7], L61H10 [8], and lobaplatin [9] were shown to be effective against esophageal cancer, lung cancer, and colon cancer, respectively, by inducing a *DFNA5*-dependent pyroptosis effect. These results have led to a new understanding of cancer chemotherapy, while indicating that *DFNA5* is a potential target for cancer treatment. However, the role of *DFNA5* in HNSCC development and progression remains unknown.

In this work, we applied a wide range of comprehensive bioinformatics tools to assess the expression levels and potential function of *DFNA5*, as well as determine its prognostic value in human HNSCC.

## Materials and methods

### Oncomine

In order to evaluate the expression of DFNA5 in different tumors, Oncomine (https://www.oncomine.org/resource/login.html) is a publicly accessible online cancer microarray database that can be used for research related to genome-wide expression analysis. We used this database to extract data on the *DFNA5* mRNA levels (log2-transformed) in HNSCC and neighboring normal tissues. The criteria for identifying a significant difference were a *P*-value <1E-4, fold change > 2, and the gene ranks in the top 10% [10].

### Timer

TIMER is a web server for the comprehensive analysis of expression pattern and tumor-infiltrating immune cells. It offers six tumor-infiltrating immune subsets precalculated for 10,897 tumors from 32 cancer types (https://cistrome.shinyapps.io/timer/) [11]. TIMER web server allows users to input function-specific parameters, with resulting figures dynamically displayed to conveniently access the tumor immunological, clinical, and genomic features.

### TISIDB

TISIDB is an integrated repository portal for tumor-immune system interactions, which can also be used for the systematic testing of molecular features of such interactions TISIDB is the most comprehensive database for tumor and immune system interactions. In TISIDB, users can cross check the gene of interest through knowledge from different platforms and obtain high quality visualization results for their publications (http://cis.hku.hk/TISIDB/) [12]. In present study, we use TISIDB to predict the prognosis evaluation.

### cBioPortal

cBioPortal includes tools for the visualization, analysis, and downloading of large-scale cancer genomics datasets. In order to regulatory network of *DFNA5*, we used cBioPortal to identify genes positively related to *DFNA5* expression in HNSCC [13].

### GEPIA

GEPIA (http://gepia.pku.cn/) is a newly developed interactive web server for analyzing the RNA sequencing expression data of 9736 tumors and 8587 normal samples from the TCGA and the GTEx projects, using a standard processing pipeline. GEPIA provides customizable functions such as tumor/normal differential expression analysis, profiling according to cancer types or pathological stages, patient survival analysis, similar gene detection, correlation analysis and dimensionality reduction analysis. In present study, GEPIA was used to analysis differential expression and survival analyses [14].

### Kaplan-Meier plotter

The Kaplan Meier plotter is capable to assess the effect of 54 k genes (mRNA, miRNA, protein) on survival in 21 cancer types. Sources for the databases include GEO, EGA, and TCGA. Primary purpose of the tool is a meta-analysis based discovery and validation of survival biomarkers. Kaplan-Meier plotter (http://kmplot.com/analysis/) was used to evaluate the potential prognostic significance of *DFNA5* [15]. The hazard ratio (HR) with a 95% confidence interval and the *P*-value (log-rank) were calculated. To research the relationship between the *DFNA5* expression level and specific clinical characteristics of HNSCC patients. Enter *DFNA5* in the website of Kaplan-Meier plotter, select HNSCC in Pan-cancer RNA-seq database, and then select the corresponding index (as shown in Table 2) in the right-hand border, click draw Kaplan-Meier plot to record the corresponding results.

### Cytoscape

Cytoscape (version 3.4.0) is an open-source bioinformatics software tool for visualizing molecular interaction networks. Its plug-in, MCODE, is used for ranking nodes in a network according to their network features [16]. Presently, we use Cytoscape to study the regulatory network of DFNA5 in HNSCC.

### David

DAVID (http://david.abcc.ncifcrf.gov/) is a tool that is widely used to reveal the biological significance of gene groups. After selecting co-expressed genes, Gene Ontology (GO) analysis was performed to assess their associated biological processes (BP), cellular components (CC), and molecular functions (MF) [17]. The Kyoto Encyclopedia of Genes and Genomes (KEGG) pathway database was used to identify biological pathways enriched with the co-expressed genes [18].

### UALCAN

UALCAN (http://ualcan.path.uab.edu) is an interactive web resource that can combine clinical data for 31 cancer types with corresponding RNA-seq data from the TCGA database. It is built on PERL-CGI with high quality graphics using javascript and CSS. As such, it can be used to analyze relative gene transcript levels between tumors and normal samples, and the relationship between these levels and clinicopathological parameters [19].

### Realtime PCR

We use HNSCC cell lines for in vitro gene expression validation, such as SCC-15 (ATCC CRL-1623), Cellosaurus HN6 (CVCL_8129), and Human oral squamous cell carcinoma (HSC-3); Human normal oral keratinocytes (NOK) as control cells. Total RNA was isolated from cell lines using TRIzol (Takara). RNA was then converted to cDNA using PrimeScript RT Master Mix (Takara). qPCR was performed on a Bio-Rad Thermal Cycler using SYBR Premix Ex Taq II (Takara) with primers against human DFNA5. For-ACATGCAGGTCGAGGAGAAGT; Rev-TCAATGACACCGTAGGCAATG. Relative expression was calculated using the delta-delta Ct method and normalized to the reference gene GADPH For-ACAACTTTGGTATCGTGGAAGG; Rev-GCCATCACGCCACAGTTTC.

### Statistical analysis

The survival curves were generated via Kaplan- Meier plots and PrognoScan database are displayed with HR and P or Cox *P*-values from a log-rank test. Gene expression data in the Oncomine database was analyzed using *p*-value, fold change, and mRNA data type. Spearman correlation analysis was used to evaluate the correlation of gene expression in TIMER, LinkedOmics and UALCAN databases. *P < 0.05, **P < 0.01, ***P < 0.001 was considered statistically significant.

The list of experiments were listed in Table 1.

**Table 1:** The list of experiments

Methods (database)	Results
Oncomine, TIMER, GEPIA, UALCAN, qRT-PCR	Expression of DFNA5
Kaplan-Meier plotter, TISIDB, and UALCAN	Prognosis value of DFNA5
cBioPortal, UALCAN, DAVID, CytoHubba	Regulatory network of DFNA5
TIMER	Infiltration of immune cells

## Results

### *DFNA5* expression is upregulated in human HNSCC

**Fig. 1:**
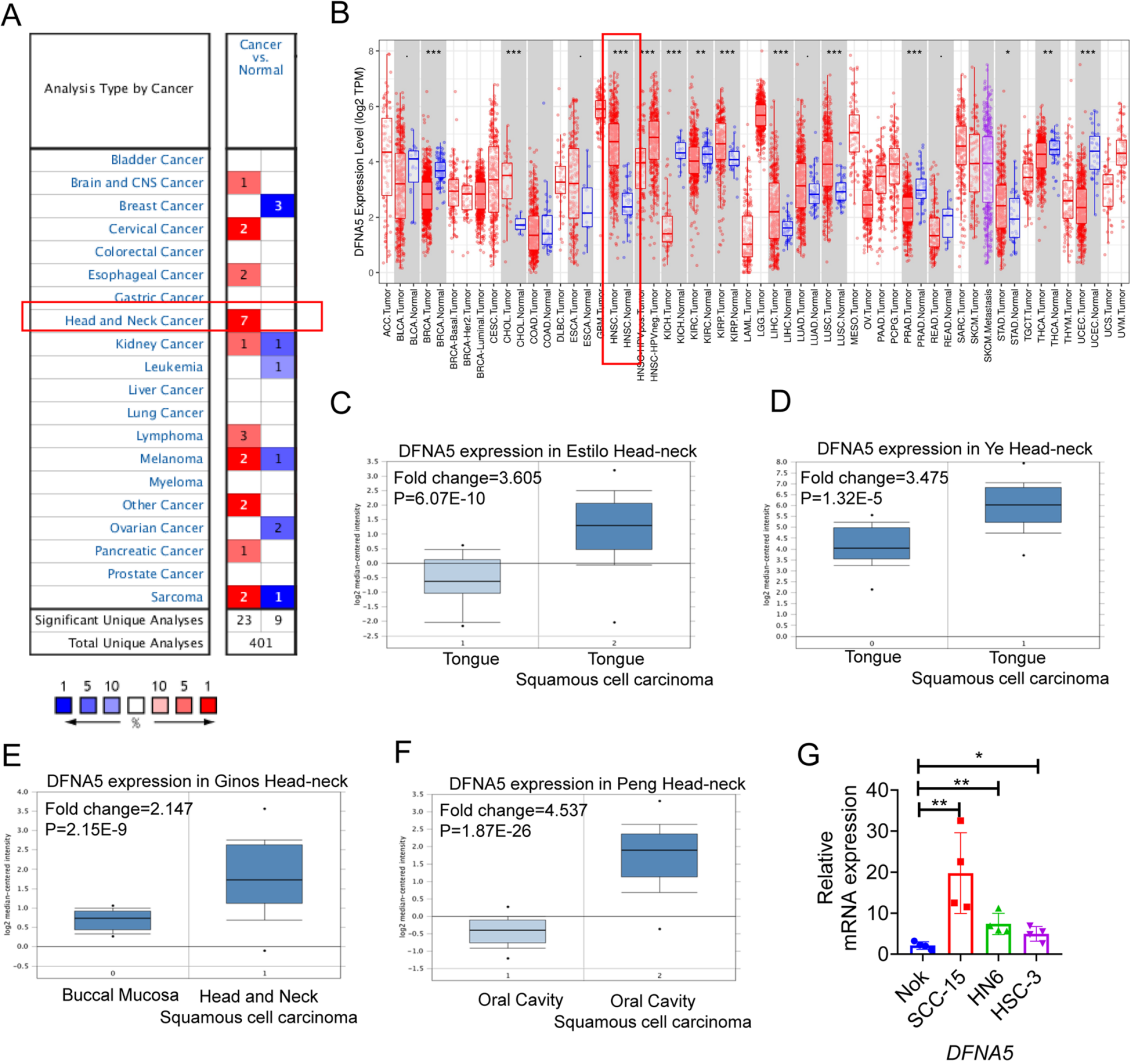
DFNA5 expression in human head and neck squamous cell carcinoma. **(A-B)** The expression level of *DFNA5* in HNSCCs and healthy tissues as calculated from the gene expression data of the Oncomine database (**A**), and TIMER database (**B**). (**C-F**) Analysis of *DFNA5* expression in different subtypes of HNSCC using gene expression data from the Oncomine database. (**G**) In vitro validation of the expression level of *DFNA5* in the HNSCC cell lines SCC-15 (ATCC CRL-1623), Cellosaurus HN6 (CVCL_8129), and human oral squamous cell carcinoma (HSC-3); human normal oral keratinocytes (NOKs) were used as control cells. *P < 0.05, **P < 0.01, ***P < 0.001

The analysis of microarray data from HNSCC and adjacent normal tissues in the Oncomine database showed that *DFNA5* expression was upregulated in HNSCC (Fig. 1A). This pattern was confirmed by the analysis of gene expression data deposited in TIMER (Fig. 1B). In addition to bulk analysis of HNSCC data, we also used Oncomine to compare *DFNA5* expression in healthy and cancer tissues from datasets corresponding to different HNSCC subtypes. These results showed that *DFNA5* upregulation is a general characteristic of HNSCC (Fig. 1C-F). Further subgroup analysis of various clinicopathological features of HNSCC patients from the TCGA database revealed that in subgroup analyses based on gender, age, race, disease stage, tumor grade, human papillomavirus infection, and nodal metastasis status, the transcription level of *DFNA5* in HNSCC patients was consistently significantly higher than that in healthy subjects (Sup Fig. 1). Furthermore, we used HNSCC cell lines for in vitro validation, demonstrating a significant increase in the level of *DFNA5* compared to that of control cells (Fig. 1G). Collectively, these results suggest that the expression of *DFNA5* may be a potential diagnostic marker of HNSCC.

### High *DFNA5* expression predicts poor prognosis in HNSCC patients

**Fig. 2:**
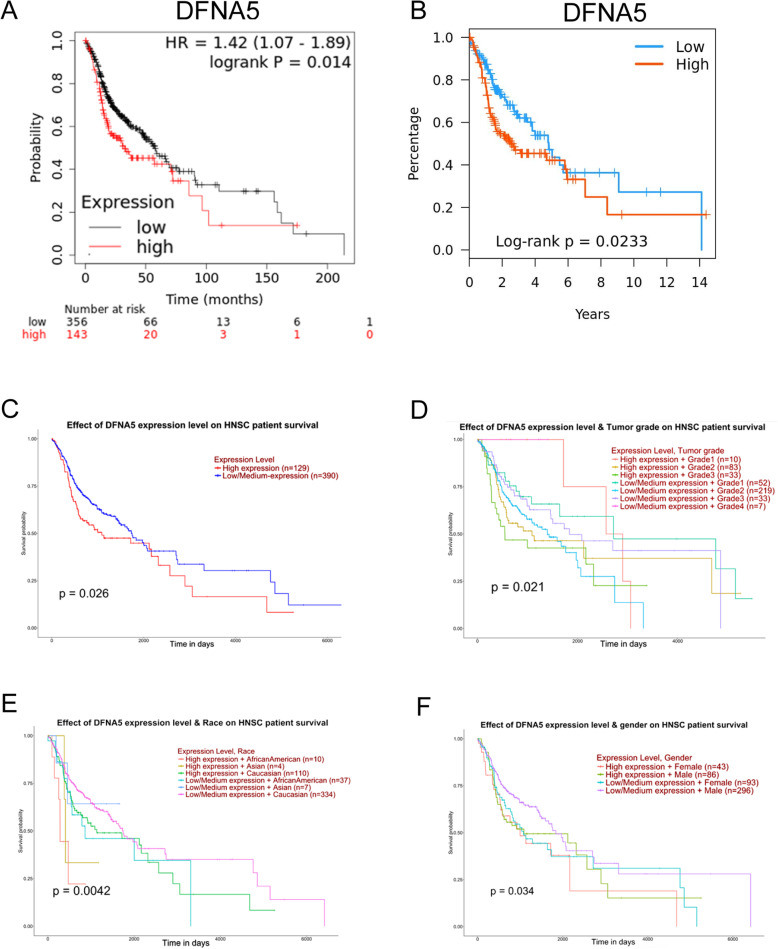
DFNA5 prognostic value in human head and neck squamous cell carcinoma. (**A-C**) Correlation between overall survival (OS) and *DFNA5* expression in patients with HNSCC as assessed using the Kaplan-Meier plotter (**A**), TISIDB (**B**), and UALCAN (**C**). (**D-F**) Correlation between OS and *DFNA5* expression for patients of different tumor grades (D), races (E), and genders (F) as assessed using UALCAN. *P < 0.05

We speculated that the expression of *DFNA5* is related to the prognosis of HNSCC patients. To test this hypothesis, we evaluated the prognostic value of *DFNA5* using the Kaplan-Meier plotter, TISIDB, and UALCAN (Fig. 2A-C, respectively). All three analyses showed that high *DFNA5* expression levels are associated with a poor prognosis in HNSCC patients. Moreover, this association was consistent regardless of tumor grade (Fig. 2D), race (Fig. 2E), or gender (Fig. 2F). To further understand this correlation and assess the potential underlying mechanism, we utilized the Kaplan-Meier plotter to explore the relationship between the *DFNA5* expression level and specific clinical characteristics of HNSCC patients. As shown in Table 2, high expression of *DFNA5* was significantly correlated with worse overall survival (OS) only in male patients (*P* = 0.041). Moreover, racial background did not have a statistically significant effect. With respect to tumor stage, *DFNA5* overexpression was associated with poorer OS in HNSCC patients at all stages except for stage 3. More importantly, we found that high expression of *DFNA5* corresponded with a worse OS in patients whose tumors were infiltrated by immune cells such as basophils, B-cells, T-cells, and macrophages (Table 2). These results suggest that *DFNA5* may be a reliable biomarker for HNSCC prognosis, at least in male patients.

**Table 2:** Correlation between DFNA5 expression and clinical prognosis in head and neck squamous cell carcinoma with different clinicopathological factors using the Kaplan-Meier plotter

Clinicopathological characteristics		OS survival		
		N	Hazard ratio	*P*-value
Gender:	Female	133	1.56 (0.95–2.55)	0.076
	**Male**	**366**	**1.43 (1.01–2.03)**	**0.041**
Race:	**White**	**426**	**0.35 (1.01–1.82)**	**0.044**
	**black/African American**	**47**	**3.4 (1.3–8.91)**	**0.0084**
	**1**	**25**	**7.29 (0.76–70.09)**	**0.044**
Stage	**2**	**69**	**0.45 (0.2–1)**	**0.043**
	3	78	1.55 (0.68–3.55)	0.29
	**4**	**259**	**1.75 (1.21–2.53)**	**0.0027**
	1	61	1.94 (0.8–4.7)	0.14
Grade:	2	298	1.29 (0.91–1.82)	0.15
	**3**	**119**	**2.25 (1.29–3.92)**	**0.0033**
	4	7		
	high	251	1.34 (0.91–1.98)	0.14
Mutation burden:	**low**	**243**	**1.95 (1.29–2.97)**	**0.0014**
	**enriched**	**243**	**1.7 (1.11–2.6)**	**0.013**
Basophils:	decreased	254	1.22 (0.83–1.79)	0.3
	**enriched**	**238**	**2.07 (1.31–3.28)**	**0.0015**
B-cells:	decreased	259	1.16 (0.77–1.73)	0.48
	**enriched**	**316**	**1.66 (1.16–2.37)**	**0.0048**
CD4+ memory T-cells:	decreased	181	1.3 (0.84–2.01)	0.24
	**enriched**	**305**	**1.55 (1.05–2.27)**	**0.025**
CD8+ T-cells:	**decreased**	**192**	**1.62 (1.04–2.51)**	**0.03**
	enriched	215	1.55 (1.05–2.27)	0.025
Eosinophils:	**decreased**	**282**	**1.53 (1.07–2.19)**	**0.02**
	enriched	205	1.49 (0.91–2.38)	0.12
Macrophages:	decreased	292	1.34 (0.93–1.94)	0.12
	**enriched**	**313**	**1.62 (1.15–2.27)**	**0.0053**
Mesenchymal stem cells:	decreased	184	1.33 (0.85–2.07)	0.21
	**enriched**	**129**	**1.54 (0.94–2.52)**	**0.082**
Natural killer T-cells:	decreased	368	1.36 (0.97–1.9)	0.077
	**enriched**	**322**	**1.43 (1.01–2.01)**	**0.04**
Regulatory T-cells:	decreased	175	1.54 (0.95–2.48)	0.074
	**enriched**	**381**	**1.53 (1.11–2.11)**	**0.0082**
Type 1 T-helper cells:	decreased	116	1.38 (0.8–2.39)	0.24
	**enriched**	**473**	**1.39 (1.04–1.85)**	**0.024**
Type 2 T-helper cells:	decreased	24	4.84 (0.8–29.1)	0.057

### *DFNA5* regulates cell adhesion in HNSCC

**Fig. 3:**
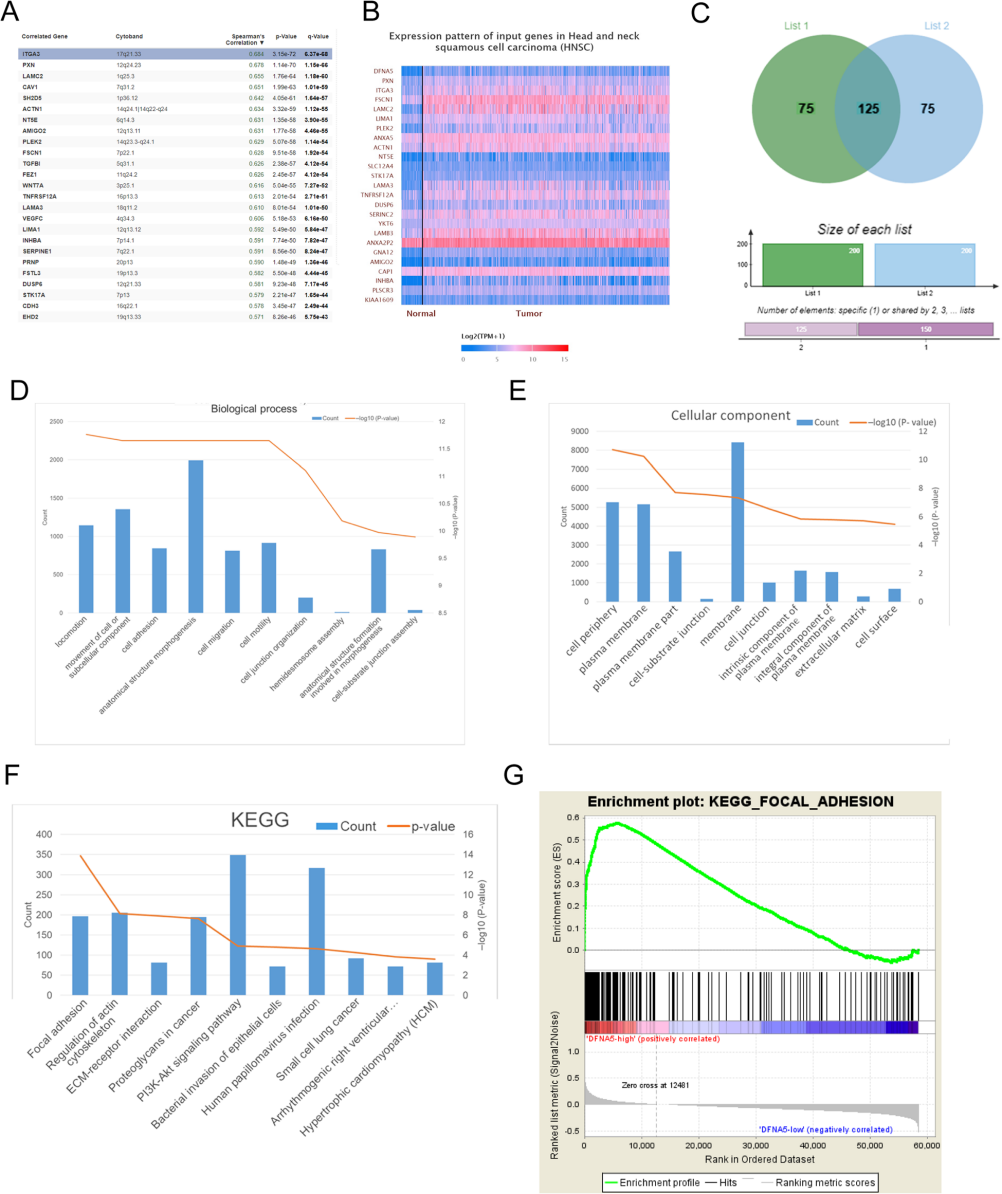
Enrichment function analyses of co-expressed genes indicating an association of DFNA5 with cell adhesion. (**A, B**) Lists of the top 200 genes positively correlated with *DFNA5* expression as determined using cBioPortal (**A**) and UALCAN (**B**). (**C**) Venn diagram of the two lists showing an intersection containing 125 genes. (**D–F**) GO enrichment in biological processes (**D**), and cellular components (**E**), for the 125 common genes. (**F**) KEGG pathway enrichment analysis of the 125 common genes. **(G)** GSEA analysis showed that the high expression group of DFNA5 was correlated with focal adhesion pathway

Lists of the top 200 genes co-expressed with *DFNA5* were created by analyzing TGCA datasets with cBioPortal (Fig. 3A) and UALCAN (Fig. 3B). Comparison of the two lists revealed 125 common co-expressed genes (Fig. 3C). DAVID was then used to perform functional annotation of these 125 genes. GO analysis indicated that these genes were mainly involved in biological processes of locomotion, cell adhesion, and cell migration (Fig. 3D), which was consistent with the enrichment in the respective cellular components and the proposed molecular functions (Fig. 3E). In addition, KEGG pathway analysis showed enrichment in pathways of focal adhesion and the interaction between the actin cytoskeleton and the extracellular matrix receptor (Fig. 3F). Meanwhile, GSEA analysis also showed that the high expression group of DFNA5 was related to focal adhesion pathway (Fig. 3G). Collectively, these data suggest an essential role of *DFNA5* in regulating cell adhesion in HNSCC.

### Identification and analysis of *DFNA5*-related hub genes

**Fig. 4:**
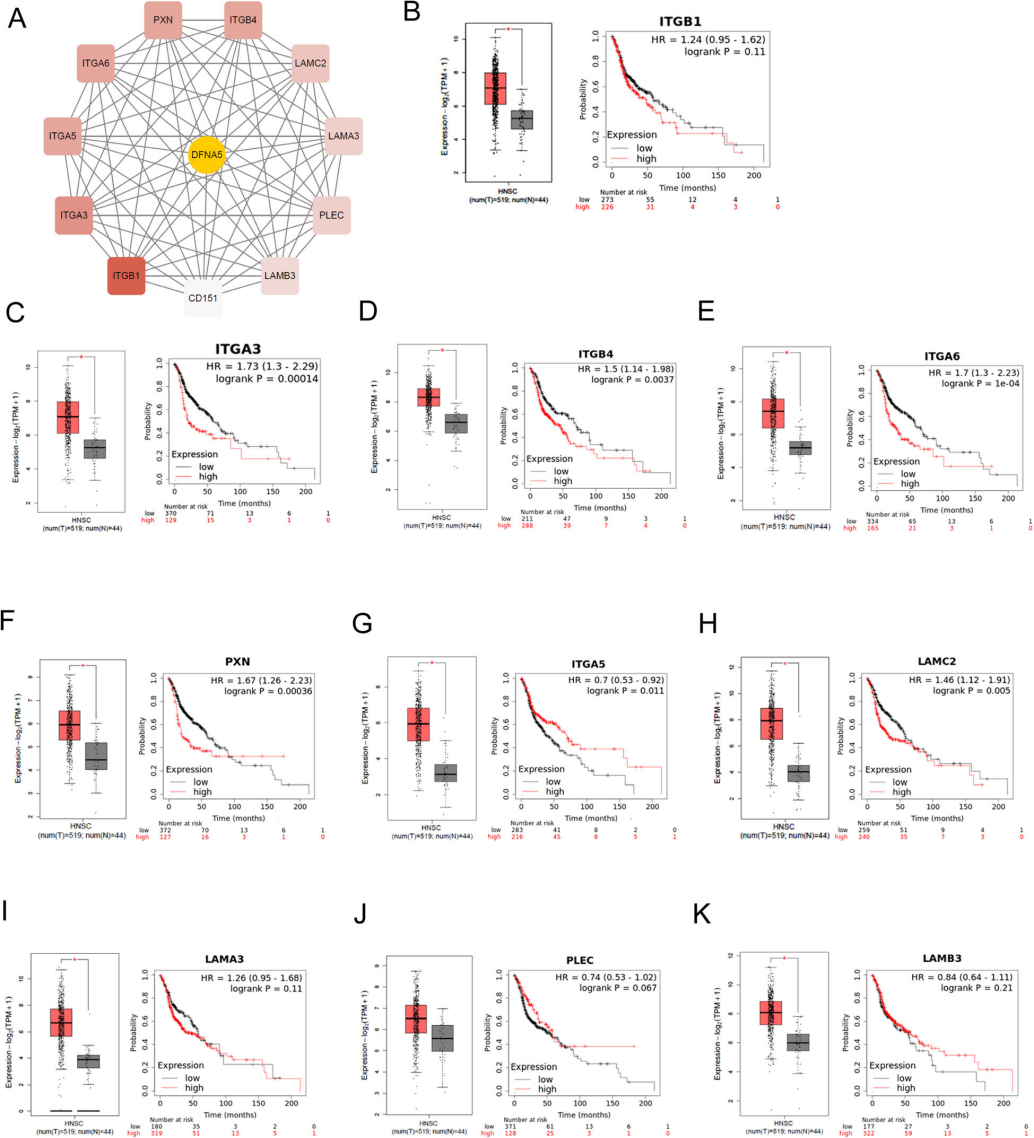
Identification and analysis of DFNA5 positively correlated hub genes. (**A**) Top 10 hub genes as identified using the cytoHubba tool kit in Cytoscape. (**B-K**) Expression level and overall survival analyses for each of the 10 identified hub genes in head and neck squamous cell carcinoma patients. *P < 0.05

CytoHubba, a Cytoscape plug-in, was used to identify potential hub genes for *DFNA5* function based on the density of maximum neighborhood component. The top 10 genes in the network included *ITGB1*, *ITGA3*, *ITGB4*, *ITGA6*, *PXN*, *ITGA5*, *LAMC2*, *LAMA3*, *PLEC*, and *LAMB3* (Fig. 4A), which were belong to these positively co-expressed genes. GEPIA was then used to analyze the expression level of each hub gene and its correlation with OS. All genes except for *PLEC* had higher expression levels in HNSCCs than in normal tissues. Moreover, high expression levels of *ITGB1*, *LAMA3*, *PLEC*, and *LAMB3* correlated with poor OS in HNSCC patients (Fig. 4B-K). These results further support that *DFNA5* mainly affects tumor progression in HNSCC by regulating cell adhesion through processes such as integrin-related molecular pathways.

### High *DFNA5* expression results in decreased lymphocyte infiltration in HNSCC

**Fig. 5:**
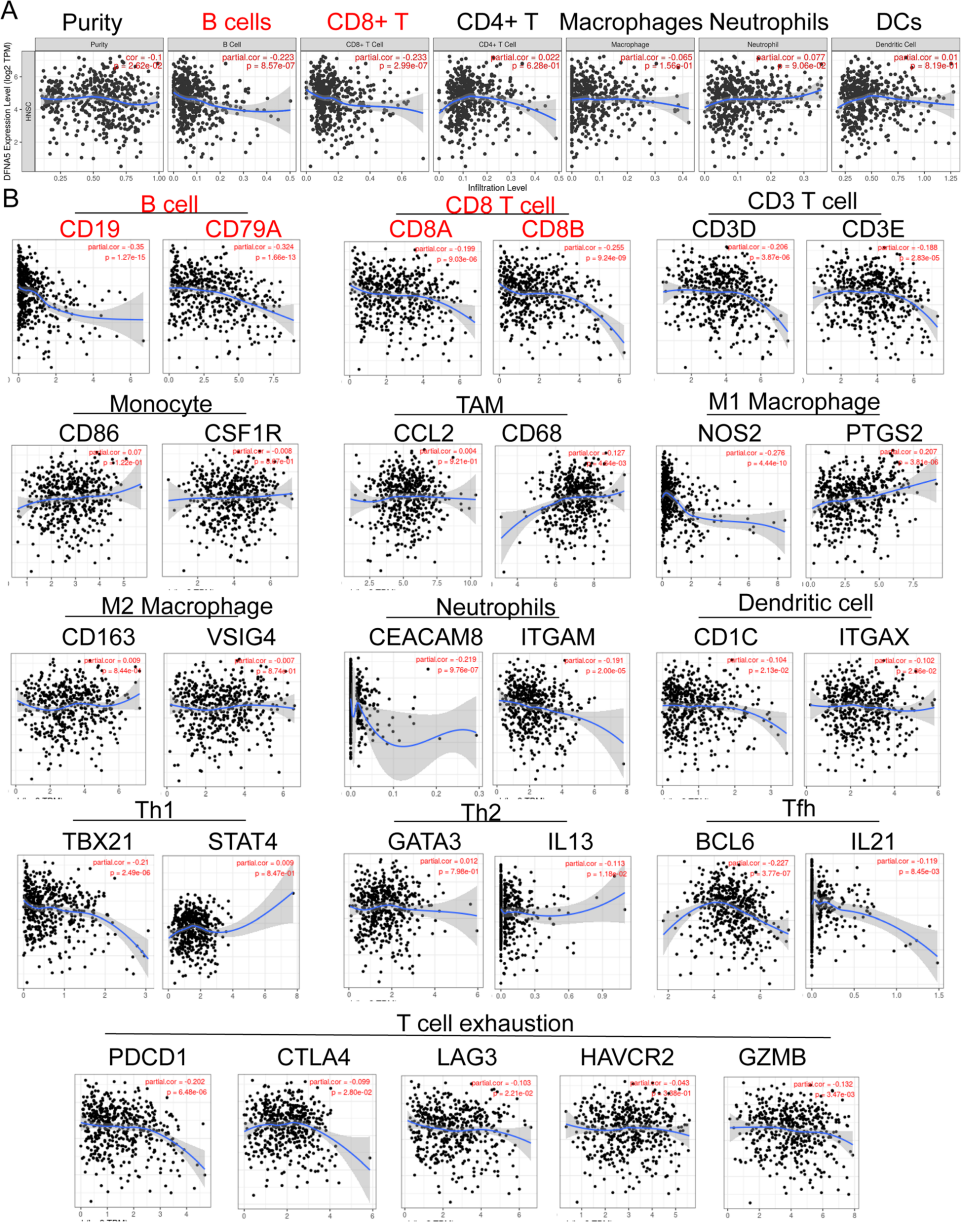
Correlation analysis of DFNA5 expression and immune cell infiltration in HNSCC. (**A**) DFNA5 expression is significantly negatively correlated with infiltrated B cells and CD8+ T cells. (**B**) Correlation between *DFNA5* and markers of various immune cells in Tumor Immune Estimation Resource (TIMER)

To further explore the function of *DFNA5* in HNSCC and its prognostic potential, we focused on the infiltration of immune cells in the HNSCC tumor microenvironment. Specifically, we used TIMER to investigate whether the expression of *DFNA5* is related to the level of immune cell infiltration. Figure 5A shows that the *DFNA5* expression level was significantly negatively correlated with the infiltration of lymphocytes in HNSCC, such as B cells (cor = − 0.223, *P* = 8.57e-07) and CD8 T cells (cor = − 0.223, *P* = 2.99e-07). Furthermore, we analyzed the correlation between *DFNA5* expression levels and various immune cell markers in HNSCC, including subsets of T cells, B cells, M1 and M2 macrophages, neutrophils, natural killer cells, and dendritic cells. The results showed that the *DFNA5* expression level was significantly negatively correlated with B cell and CD8 T cell markers (Fig. 5B). We conclude that a high level of *DFNA5* expression significantly correlates with decreased tumor local lymphocyte infiltration, which is an important negative prognostic factor in HNSCC patients.

## Discussion

HNSCCs are aggressive cancers originating from the epithelial mucosa of the digestive tract [20, 21]. Although many studies have focused on the macro-genomic differences underlying the heterogeneity of these cancers, the survival rate of patients has not improved in the past decade [22]. Here, we showed that *DFNA5* exerts oncogenic effects in HNSCC, as it is consistently overexpressed in cancer tissues and is an indicator of poor prognosis. Moreover, GO and KEGG pathway analyses showed that the upregulation of *DFNA5* in HNSCC mainly affects cell adhesion by regulating processes such as integrin binding, and GSEA prediction shows the same results (Fig. 3G). These results are consistent with the fact that two cell-to-cell adhesion genes that act as tumor suppressors, namely *CTNNA2* and *CTNNA3*, are frequently mutated in laryngeal carcinoma [23–25]. These adhesion proteins and their related pathways provide new candidate targets for formulating novel therapeutic strategies.

This study suggests that *DFNA5* expression negatively correlated with lymphocyte infiltration in HNSCC patients. Changes in the immune system of HNSCC patients suggest that tumorigenesis is a comprehensive immunosuppressive process [26]. In the peripheral blood, the overall number of white blood cells in patients with HNSCC is decreased, and inhibitory T cells (Treg) become dominant among them [27]. Moreover, tumor-infiltrating lymphocytes (TILs) have been detected in many types of solid tumors, including HNSCCs. In HNSCC patients, TILs have stronger anti-cancer activity than peripheral blood Treg cells [28, 29]. Recent studies have shown that B cells and plasma B cells located in tumors or the tumor-draining lymph nodes play an important role in the formation of anti-tumor immune responses [30]. Moreover, T cells and B cells interact and coordinate their selection, specialization, and clonal expansion in tumor-associated tertiary lymphoid structures [31, 32]; the resulting plasma B cells are crucial to the anti-tumor immune response. As TILs are a major factor in this process, it comes as no surprise that their immunoprofile is a prognostic marker of disease-specific survival [33]. For example, in colorectal cancer, higher CD8/CD4 ratios are associated with longer disease-free survival [34]. We found that *DFNA5* expression was significantly negatively correlated with lymphocyte infiltration, especially of B cells and CD8+ T cells, suggesting that *DFNA5* overexpression may exert its oncogenic function by hindering the anti-tumor immune response.

Notably, in contrast to our findings for HNSCC, *DFNA5* expression is suppressed in many cancers, and reduced *DFNA5* levels are associated with decreased survival in patients with breast cancer, suggesting that *DFNA5* might be a tumor suppressor. In fact, recently study determined that the expression level *DFNA5* in breast cancer patients was significantly decreased [35]; therefore, we speculate that *DFNA5* plays a different role in different tumor types.

In conclusion, this is the first study to report *DFNA5* as a new biomarker for HNSCC. More importantly, our results suggest an underlying mechanism for the oncogenic function of *DFNA5*, namely the reduction of immune cell infiltration through the modulation of cell adhesion. With further understanding of its functional scope, *DFNA5* may become an effective tool for the diagnosis and treatment of HNSCC, and may help to make biomarker therapy a promising therapeutic option.

## Supplementary Information

**Additional file 1 Sup Fig. 1** DFNA5 transcription analysis in sub groups of patients with HNSCC.

## Data Availability

The data used to support the findings of this study are available from the corresponding author upon reasonable request. Direct web links of datasets about; Oncomine: http://oncomine.org; UALCAN: http://ualcan.path.uab.edu; The Human Protein Atlas: https://www.proteinatlas.org; cBioPortal: http://cbioportal.org; LinkedOmics: http://linkedomics.org; TIMER: https://cistrome.shinyapps.io/timer/; HCCDB: http://lifeome.net/database/hccdb/home.html.
