# Supplementary material for: Prognostic role of DFNA5 in head and neck squamous cell carcinoma revealed by systematic expression analysis
Source: BMC Cancer. 2021 Aug 25;21:951. doi: 10.1186/s12885-021-08692-w (PMC8390204; doi:10.1186/s12885-021-08692-w)
Supplement: Supplementary file 1 — Additional file 1 Sup Fig. 1 DFNA5 transcription analysis in sub groups of patients with HNSCC. [file 12885_2021_8692_MOESM1_ESM.pptx]

## Slide 1
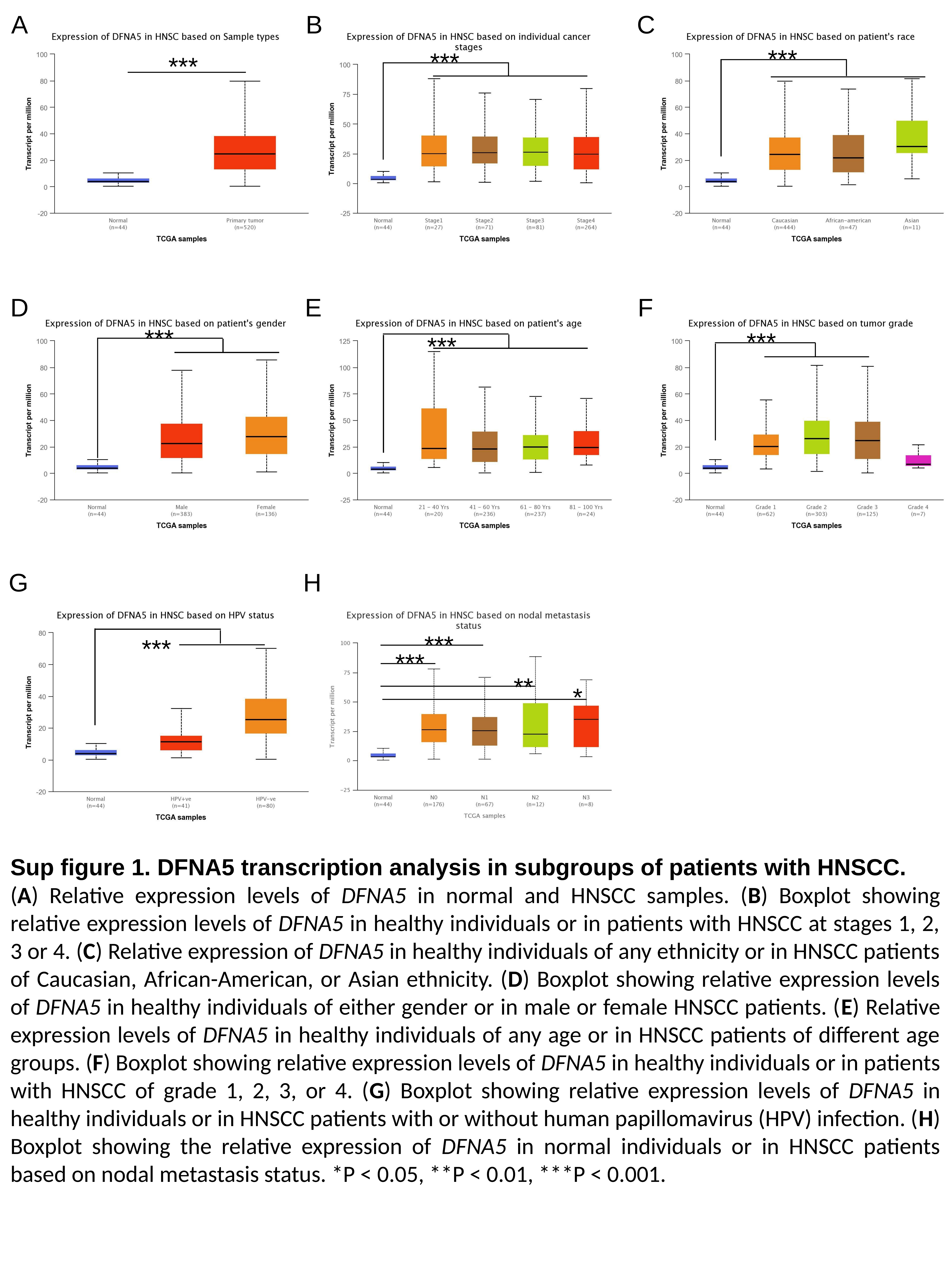

A
B
C
***
***
***
D
E
F
***
***
***
G
H
***
***
***
**
*
Sup figure 1. DFNA5 transcription analysis in subgroups of patients with HNSCC.
(A) Relative expression levels of DFNA5 in normal and HNSCC samples. (B) Boxplot showing relative expression levels of DFNA5 in healthy individuals or in patients with HNSCC at stages 1, 2, 3 or 4. (C) Relative expression of DFNA5 in healthy individuals of any ethnicity or in HNSCC patients of Caucasian, African-American, or Asian ethnicity. (D) Boxplot showing relative expression levels of DFNA5 in healthy individuals of either gender or in male or female HNSCC patients. (E) Relative expression levels of DFNA5 in healthy individuals of any age or in HNSCC patients of different age groups. (F) Boxplot showing relative expression levels of DFNA5 in healthy individuals or in patients with HNSCC of grade 1, 2, 3, or 4. (G) Boxplot showing relative expression levels of DFNA5 in healthy individuals or in HNSCC patients with or without human papillomavirus (HPV) infection. (H) Boxplot showing the relative expression of DFNA5 in normal individuals or in HNSCC patients based on nodal metastasis status. *P < 0.05, **P < 0.01, ***P < 0.001.
